# Near-Field Microwave Sensing for Chip-Level Tamper Detection

**DOI:** 10.3390/s25134188

**Published:** 2025-07-05

**Authors:** Maryam Saadat Safa, Shahin Tajik

**Affiliations:** Electrical and Computer Engineering Department, Worcester Polytechnic Institute, Worcester, MA 01609, USA; msafa@wpi.edu

**Keywords:** complementary split-ring resonator (CSRR), near-field sensing, tamper detection, hardware Trojans, physical layer security, scattering parameters, impedance characterization

## Abstract

Stealthy chip-level tamper attacks, such as hardware Trojan insertions or security-critical circuit modifications, can threaten modern microelectronic systems’ security. While traditional inspection and side-channel methods offer potential for tamper detection, they may not reliably detect all forms of attacks and often face practical limitations in terms of scalability, accuracy, or applicability. This work introduces a non-invasive, contactless tamper detection method employing a complementary split-ring resonator (CSRR). CSRRs, which are typically deployed for non-destructive material characterization, can be placed on the surface of the chip’s package to detect subtle variations in the impedance of the chip’s power delivery network (PDN) caused by tampering. The changes in the PDN’s impedance profile perturb the local electric near field and consequently affect the sensor’s impedance. These changes manifest as measurable variations in the sensor’s scattering parameters. By monitoring these variations, our approach enables robust and cost-effective physical integrity verification requiring neither physical contact with the chips or printed circuit board (PCB) nor activation of the underlying malicious circuits. To validate our claims, we demonstrate the detection of various chip-level tamper events on an FPGA manufactured with 28 nm technology.

## 1. Introduction

Integrated circuits (ICs) are essential to modern electronic systems, powering applications across consumer electronics, defense and aerospace, medical devices, smart grids, transportation, and data centers. To meet growing demand and production efficiency, the semiconductor supply chain has evolved into a global network, with components manufactured across diverse locations. This globalization of the semiconductor design and manufacturing process has introduced serious security concerns, particularly the risk of hardware Trojan (HT) insertion and the introduction of counterfeit components. Such threats can compromise the functional integrity and reliability of integrated circuits throughout their operational lifetime. In particular, malicious alterations to the designs of application-specific integrated circuits (ASICs) and field-programmable gate arrays (FPGAs) can jeopardize the security of critical systems. While many of these threats fall under the category of hardware Trojans, other forms of tampering can still pose significant risks to system security. As a result, a wide range of detection techniques have been developed to identify both HTs and other types of unauthorized modifications. However, conventional physical inspection methods can detect such tamper events, but they are often destructive, expensive, and impractical for large-scale post-silicon validation. The existing non-invasive side-channel techniques also suffer from noise, resulting in low-confidence tamper detection.

Tamper detection generally faces two primary challenges. First, many HTs are designed to remain dormant under normal operating and testing conditions, making their detection difficult. While early HTs could often be triggered through logical testing, more recent designs are significantly more stealthy. These Trojans are activated only under particular and rare conditions, such as specific temperature ranges, supply voltages, or clock frequencies, or after a defined sequence of events. The second challenge stems from the detection methods themselves, as many require at least some level of direct access or interaction with the chip, which may not always be feasible, especially in deployed or third-party systems.

To address the challenge of detecting dormant Trojans, a non-invasive technique based on electromagnetic (EM) backscattering has been proposed [1,2,3,4]. In this method, EM waves at specific frequencies are injected into the chip using a transmitting antenna, while a receiving antenna captures the reflected signals modulated by the chip’s internal switching activity. The underlying assumption is that static impedance changes from added circuitry subtly alter the die’s overall impedance, which in turn affects the switching behavior and current consumption of neighboring circuits. These variations modulate the backscattered signal, particularly in its harmonic content. Detecting such subtle changes requires advanced signal processing techniques and often machine learning applied across multiple measurements, along with careful tuning of carrier and modulation frequencies specific to the target technology and circuit design. Moreover, the experimental setup must be meticulously engineered to minimize interference from RF noise, temperature variations, and ambient wireless signals, making the implementation both technically demanding and highly sensitive to environmental conditions. These challenges are further compounded by the need for sophisticated and costly measurement equipment.

Another approach for detecting dormant Trojans and counterfeit components involves characterizing the impedance of the system’s power delivery network (PDN) [5,6,7]. Physical modifications to the PCB or chip can alter the equivalent impedance of the PDN, and analyzing its behavior across a range of frequency bands may reveal violations of the system’s physical integrity. This analysis typically relies on extracting scattering (S-) and impedance (Z-) parameters. The most conventional technique employs a vector network analyzer (VNA) connected via SMA connectors to designated PDN-accessible points on the PCB. However, in many practical cases, such access points are unavailable, limiting the applicability of this method. Alternatively, on-chip impedance sensors can be used, but they require modifications to the chip’s internal configuration [8].

To address the second challenge, researchers have proposed semi- and fully invasive techniques such as laser-assisted probing and high-resolution imaging (e.g., scanning electromicroscopy) [9,10,11]. Although passive and non-invasive side-channel analysis techniques [12,13,14] offer a lower-risk alternative, they suffer from limited spatial resolution and are often incapable of detecting stealthy or dormant tampering. In contrast, invasive methods provide greater precision and visibility into internal structures, making them more powerful for detecting subtle or concealed hardware Trojans without the need for connection to the chip. Some detection approaches incorporate additional on-chip measurement circuitry to facilitate post-silicon Trojan detection, but this added circuitry increases die area, manufacturing cost, and power consumption, rendering such techniques incompatible with legacy systems [15]. Nevertheless, these methods are often time-consuming, may require invasive package preparation, and can be destructive, thereby limiting their scalability for widespread deployment. Despite the advancements in Trojan detection techniques, a high-precision method capable of identifying dormant Trojan circuits without requiring physical contact or direct access to the chip under test remains absent.

Motivated by the limitations discussed above, the following research question arises: can a single contactless sensor, solely by monitoring the chip’s PDN impedance in the frequency domain, reliably detect a wide range of tamper events across different classes and sizes, without requiring the triggering or activation of any part of the circuit under test?

**Figure 1:**
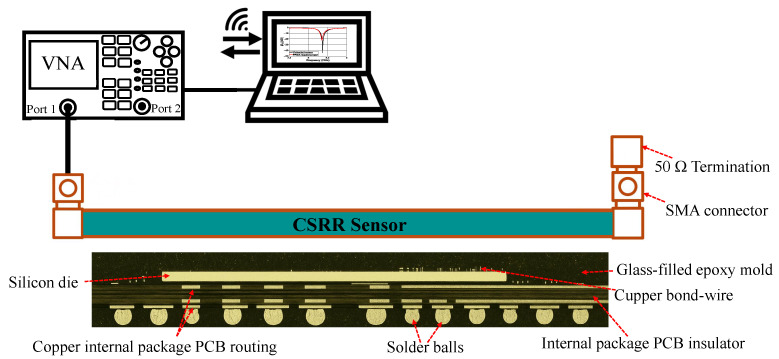
A cross-sectional view of the proposed tamper detection setup. The CSRR sensor is positioned above the FPGA at a specific distance and connected to a vector network analyzer (VNA) to measure the $S_{11}$ parameter. FPGA image based on [16].

**Contribution**: In this work, we present a fully contactless method for detecting chip-level tampering that requires no physical access to the chip or PCB and does not depend on triggering Trojans or activating parts of potentially malicious circuits. The approach repurposes a complementary split-ring resonator (CSRR) sensor, which is typically used for material characterization by analyzing shifts in its resonance frequency and quality factor (*Q*) when a material is placed in its electromagnetic near-field region and perturbs the local electromagnetic fields. The main idea is that any form of physical tampering, regardless of its type, trigger method, or intended effect, will inevitably change the chip’s PDN impedance. When the chip is positioned within the near field of the CSRR sensor, the chip’s PDN impedance perturbs the sensor’s local electromagnetic field, resulting in measurable changes to the sensor’s impedance. Figure 1 shows the cross-section of the CSRR sensor positioned above the chip’s surface, as well as the connection of the CSRR to the VNA. Instead of relying on absolute impedance values, the method detects anomalies by monitoring deviations from a trusted baseline. By sweeping across a range of frequencies and measuring the sensor’s scattering parameters (S-parameters), we capture these subtle shifts, enabling reliable, non-invasive detection of tampering across a wide variety of attack scenarios.

## 2. Background

### 2.1. CSRR-Based Sensor

Complementary split-ring resonator (CSRR) sensors are specially designed electromagnetic structures that offer a simple yet powerful way to measure the electrical and magnetic properties of materials. What makes them unique is their ability to focus electric and magnetic fields in separate regions of the sensor. When a material under test (MUT) is placed in the electric field-dominant region of a CSRR sensor, changes in resonant frequency and quality factor primarily indicate variations in permittivity. Conversely, when the MUT is positioned in the magnetic field-dominant zone, the sensor response predominantly reflects changes in permeability. This dual sensitivity enables accurate, broadband characterization of advanced materials, especially those that respond to both electric and magnetic fields (magneto-dielectrics). These effects arise because the MUT perturbs the sensor’s localized electromagnetic fields, altering how it stores and exchanges energy. This alters the sensor’s impedance, leading to measurable shifts in its resonance behavior, quality factor, and overall reflection coefficient ($S_{11}$). The resulting changes in the sensor’s reflection coefficient provide a clear signature of the material’s electromagnetic properties, forming the basis for precise, non-contact material analysis [17]. The sensitivity of a CSRR sensor quantifies its ability to detect changes in the electromagnetic properties of the material under test, typically permittivity (ε) or permeability (μ). It is commonly defined as the change in resonance frequency or quality factor in response to variations in the MUT. For example, in the case of changes in permittivity, the sensitivity can be expressed as follows:

(1) $$S_{f}=\frac{\Delta f_{r}}{\Delta \varepsilon }$$

(2) $$S_{Q}=\frac{\Delta Q}{\Delta \varepsilon }$$

where $f_{r}$ is the resonance frequency, *Q* is the quality factor, and Δ indicates the corresponding change due to the MUT.

CSRR sensors are now being used in many fields beyond material characterization. For instance, CSRR structures are widely used in filter design to achieve compact, high-selectivity bandstop and bandpass filters due to their strong resonance behavior [18,19]. In biomedicine, they help monitor tissue properties, detect glucose levels, and analyze small volumes of biological fluids [20,21,22]. In industry, they are useful for detecting moisture in materials, checking for uniformity during production, and monitoring fluid behavior in micro channels [23,24]. Due to their contactless operation and high-resolution sensitivity to small changes, CSRR sensors are also helpful for structural health monitoring, such as detecting hidden cracks in buildings or aircraft components [25].

### 2.2. Electromagnetic Behavior of CSRR Structures

The fundamental operating principle of CSRR-based sensors is to monitor shifts in the resonant frequency when a MUT is introduced into the sensing region. In the absence of a MUT, the electric and magnetic fields ($E_{0}$ and $H_{0}$) stored within the CSRR structure remain in equilibrium. However, when the MUT interacts with the CSRR, it perturbs these stored fields and generates new ones ($E_{1}$ and $H_{1}$), resulting in a shift in the resonant frequency. The parameters ($\epsilon_{0}$ and $\mu_{0}$) represent the dielectric constant and magnetic permeability of free space, respectively, characterizing the intrinsic electromagnetic properties. The change in resonant frequency ($\Delta f_{r}$) is related to the variations in dielectric constant (Δϵ), magnetic permeability (Δμ), and the effective volume (Δv) of the MUT, as described by the following relation:

(3) $$\frac{\Delta f_{r}}{f_{r}}=\frac{\int_{v}\left(\Delta \epsilon E_{1}\cdot E_{0}+\Delta \mu H_{1}\cdot H_{0}\right)\;dv}{\int_{v}(\epsilon_{0}|E_{0}{|}^{2}+\mu_{0}|H_{0}{|}^{2})\;dv}$$

At its resonant frequency, a CSRR exhibits stronger electric field localization compared to a conventional isotropic split-ring resonator (SRR) structure. When two CSRRs are placed in close proximity, electromagnetic coupling occurs between them through mutual induction and near-field energy exchange. This coupling facilitates the transfer of electromagnetic energy between the two resonators, enabling interaction-sensitive behaviors that can be exploited for sensing [26,27,28]. In this work, due to the stealthy nature of the attacks, the resulting changes in the chip’s PDN impedance are minimal, leading to subtle shifts in the sensor’s resonance frequency and quality factor. As a result, we focus on monitoring variations in the scattering parameter, specifically the phase of the reflection coefficient, across the entire frequency band. The parameter $S_{11}$ serves as an alternative representation of impedance, since the sensor impedance, $Z_{\mathrm{Sensor}}$, is related to the reflection coefficient by $Z_{\mathrm{Sensor}}=Z_{0}\frac{1\;+\;S_{11}}{1\;-\;S_{11}}$, where $Z_{0}$ is the characteristic impedance of the cables connected to the vector network analyzer (VNA) [29].

In traditional material characterization, the objective is to measure changes in the permittivity or permeability of the material under test. However, in our case, the material under test is a chip along with its package and internal components. Rather than extracting changes in permittivity, our focus is on detecting variations in the overall impedance of the chip.

**Figure 2:**
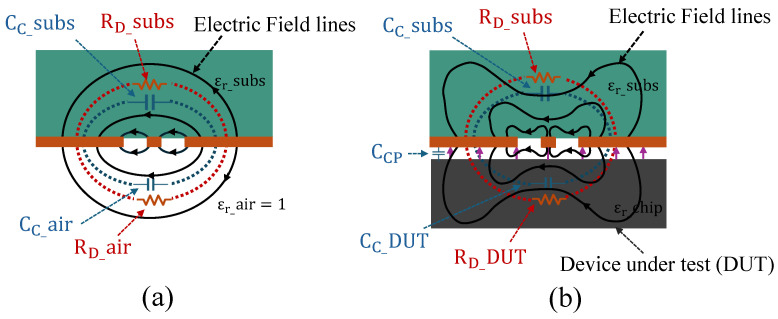
Cross-sectional view of the CSRR sensor slot region, illustrating electric field distributions and their contributions to the overall capacitance and dielectric resistance. (**a**) Electric field lines (black solid lines), along with the equivalent coupling impedance model, illustrate the formation of capacitive and resistive elements in the CSRR sensor in the absence of a chip. (**b**) Electric field lines of the CSRR sensor (black solid lines) along with the emanated electric fields from the powered-on chip under test (purple solid lines), illustrating the perturbation of the electric field on the sensor and modified impedance model due to the chip’s presence, inspired by [30].

In Figure 2, a cross-sectional view of the CSRR structure is shown, illustrating how the placement of the chip in the near-field region influences the overall electric field distribution of the sensor and its impedance. The coupling capacitance, $C_{C}$, consists of the substrate capacitance $C_{C\_\mathrm{subs}}$ and the contribution from the air region $C_{C\_\mathrm{air}}$. Similarly, the dielectric loss component $R_{D}$ includes both the substrate losses and the losses associated with the MUT. In the absence of the chip, this is represented as $R_{D\_\mathrm{air}}$, which approaches *∞* since air can be considered a perfect insulator. The relative permittivity of the sensor substrate is denoted by $\varepsilon_{r\_\mathrm{subs}}$, which in this case is 3.48. The air dielectric constant is represented by $\varepsilon_{r\_\mathrm{air}}$, and $\varepsilon_{r\_\mathrm{chip}}$ denotes the effective permittivity of the chip. This value accounts for the combined contributions of various materials within the chip, including metal interconnects, silicon, and packaging materials. Note that in our case, we did not explicitly include $L_{c}$ because the material under test exhibits negligible magnetic properties, making the inductive coupling insignificant. When the chip under test is powered on and positioned within the near-field region, the electric field lines are modified, and both the capacitance and resistance values are altered due to coupling with the chip’s impedance and the contribution of its emanated electric field.

### 2.3. Sources and Mechanisms of Chip PDN Impedance Variation

The impedance of a chip is influenced by a complex interplay of resistive and capacitive elements within its power delivery network. Key resistive sources include the intrinsic resistance of the power grid metal layers, the channel resistance of transistors, the resistance at the transistor gate, and the contact resistances associated with n-well and p-substrate regions. The resistive paths within the chip, collectively represented as $R_{die}$, influence how voltage drops and power is lost as current flows through the die. When design changes occur, such as adjustments to placement or routing, these paths can shift, resulting in changes to the chip’s PDN overall impedance. Moreover, the electrical characteristics of the substrate itself, including its impurity level profile and parasitics, further affect the resistive and reactive behavior of the PDN impedance.

**Figure 3:**
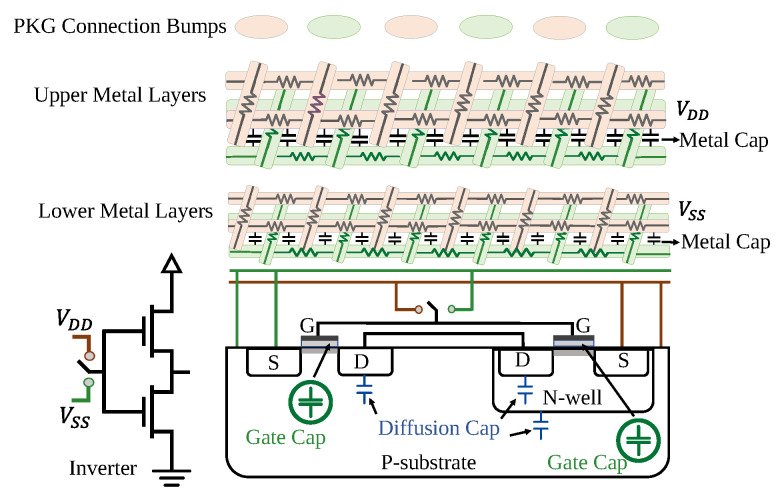
The physical representation of a CMOS inverter cross-section and the locations of different types of on-die capacitors. The black capacitors show the capacitance of metal lines, the blue ones show the p-n diode junction diffusion capacitance, and the capacitance shown in green color corresponds to non-switching gate capacitance, inspired by [31].

On the capacitive side, the dominant contributor to on-die capacitance ($C_{die}$) is the gate capacitance ($C_{g}$) of powered-on transistors, as these form active channels under the gate and thus directly couple to the power grid. In contrast, powered-off transistors do not significantly influence $C_{die}$, resulting in a capacitance that is highly dependent on the device’s power state. Additional capacitive components include the metal capacitance ($C_{d}$), associated with the power and ground mesh structures in the metal layers, and the diffusion capacitance ($C_{d}$) from p-n diode junctions. While $C_{m}$ and $C_{d}$ contribute less significantly to the total $C_{die}$, their values are influenced by metal density, layer geometry, and substrate material permittivity. Importantly, any structural change in the chip, such as logic block relocation, routing alteration, or power mesh thinning, can shift the capacitance distribution and thus modify the chip’s impedance profile, see Figure 3.

All non-switching, powered-on transistors significantly contribute to the gate capacitance ($C_{g}$) of the chip’s PDN, as an active channel forms beneath the gate, coupling it to the circuit. In contrast, powered-off transistors lack this conductive channel and therefore have a negligible impact on the on-die capacitance ($C_{die}$). When the device is powered off, the gate capacitance effect is minimal; however, upon activation, $C_{g}$ rapidly becomes the dominant component of $C_{die}$. Any modification to the chip’s design, such as through tampering or changes in layout, can alter the distribution and value of $C_{g}$, depending on the nature, size, and location of the affected regions. These variations reshape the equivalent impedance of the chip [31].

### 2.4. Electrostatic Behavior in Clock-Halted FPGAs

When a chip is operating and the clock is halted, the dynamic switching activity ceases, effectively eliminating time-varying currents associated with logic transitions. However, the chip still draws a constant supply current due to leakage currents, biasing circuits, and any static load from powered-on transistors. This static current results in a stable distribution of charge across the chip’s PDN, which in turn establishes quasi-static electric fields. Unlike high-frequency switching transients, which generate both electric and magnetic field components, the halted-clock scenario leads to a predominantly electrostatic field environment. This condition provides a useful regime for characterizing the FPGA’s steady-state impedance profile and associated electromagnetic emissions without the interference from clock-driven activity. The spatial distribution of these quasi-static electric fields depends on the physical layout of the power grid, the locations of active transistors, and the impedance characteristics of the PDN [32].

## 3. Methodology

### 3.1. Threat Model

For our threat model, we assume that the adversary can tamper with the internal design of an ASIC or FPGA prior to verification. Tampering includes adding/removing logic gates to/from the design, changing the substrate material, modifying the placement and routing of the design without any logic addition/removal, or all of the above. Tampering can be performed to break part or all of a system, weaken its security, or steal private information. We assume that the verifier has neither control over the design nor access to internal test circuitry, and instead relies on a golden sample as a trusted reference for comparison. Prior to testing either genuine or tampered circuits, the verifier performs a calibration procedure using the same measurement setup to eliminate the effects of cables and other uncertainties. However, we assume the verifier can halt the clock signal and freeze the chip in a specific state. Finally, the verifier should be able to hold the sensor at a fixed distance of a few mm above the package to measure the impedance signature.

### 3.2. Tamper Detection Using CSRR Sensors

As described in Section 2, tamper events alter the on-chip impedance, as explained from the perspective of the die’s PDN equivalent circuit model. When the sensor is positioned close to the chip, it operates within the reactive near-field region, where mutual coupling and reactive field effects dominate. In this condition, standard far-field approximations become invalid, and precise modeling of localized field perturbations is required. These perturbations can result from small structural changes (e.g., circuit modifications within the chip) or dielectric inhomogeneities.

Changes in the chip’s PDN impedance perturb the sensor’s near electromagnetic field, resulting in local field interactions that lead to an increase or decrease in its effective capacitance. Additionally, static electric fields emanated by the static state of a powered chip can interact constructively or destructively with the sensor’s near field, altering the energy stored in the sensor and consequently changing its impedance. Although magnetic coupling or changes in current flow can influence inductance in general, they are not relevant in our case, as the clock is held constant and electrical current variations are negligible. Hence, only electric field interactions modify the distribution of the sensor’s electromagnetic fields, which consequently lead to changes in its complex value impedance. In particular, changes in the reactance (the imaginary part of the impedance) shift the sensor’s resonance frequency and affect the measured $S_{11}$ parameter [33,34,35]. The relationship between the sensor’s resonant frequency and its equivalent lumped parameters and the coupling impedance is given by

(4) $$f_{r}=\frac{1}{2\pi \sqrt{\left(L_{CSRR}+L_{c}\right)(C_{CSRR}+C_{c}})}$$

where $L_{CSRR}$ and $C_{CSRR}$ represent the equivalent inductance and capacitance of the CSRR structure, respectively, while $L_{c}$ and $C_{c}$ denote the equivalent coupling inductance and capacitance between the sensor and the chip.

Overall, analyzing the scattering signatures across different frequency bands reveals that variations in the phase of the reflection response ($∠S_{11}$) are more pronounced than variations in amplitude. Furthermore, at higher frequencies, $∠S_{11}$ response exhibits lower noise compared to the amplitude component, making it a more sensitive indicator for detecting tampering events.

**Figure 4:**
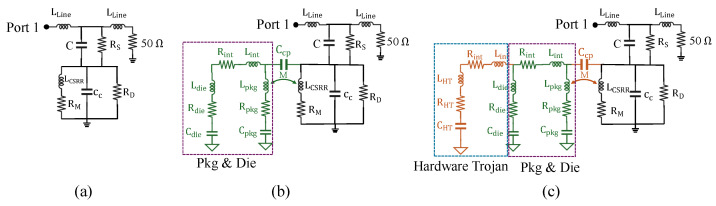
(**a**) Equivalent circuit model for CSRR sensor without the presence of a chip, inspired by [30,36,37]. (**b**) Interaction between the sensor and the chip when the chip is placed in the sensor’s near-field electromagnetic region, altering the field distribution and resulting in capacitive ($C_{\mathrm{cp}}$) and inductive (*M*) coupling between their impedance networks. (**c**) Effect of a hardware Trojan on the chip’s impedance and its influence on the sensor response.

Figure 4 shows how changes in the impedance of the chip’s PDN affect the sensor’s impedance by analyzing the equivalent impedance through circuit-level evaluation. In Figure 4a, we illustrate the equivalent circuit model of the CSRR sensor in the absence of the chip. In this model, $L_{\mathrm{Line}}$ represents the inductance of the microstrip transmission line, while *C* denotes the coupling capacitance between the line and the CSRR structure. The series resistance $R_{S}$ models dielectric losses in the substrate beneath the transmission line. The CSRR itself is modeled by an inductance $L_{\mathrm{CSRR}}$ and a capacitance $C_{C}$, with $R_{M}$ representing ohmic (metallic) losses in the resonator and $R_{D}$ accounting for dielectric losses in both the substrate and the material under test. Port 1 is connected to a VNA for S-parameter measurements, while the opposite side is terminated with a matched 50 Ω load.

In Figure 4b, the sensor is placed in the near-field region of the chip. The chip is modeled as a combination of its package and die impedances. The package is represented by the equivalent components $L_{\mathrm{pkg}}$, $R_{\mathrm{pkg}}$, and $C_{\mathrm{pkg}}$, while the die is modeled using $L_{\mathrm{die}}$, $R_{\mathrm{die}}$, and $C_{\mathrm{die}}$. $L_{\mathrm{int}}$ and $R_{\mathrm{int}}$ represent the inductance and resistance of the interconnects and traces. When the sensor is positioned above the chip, the impedance of the chip’s various PDNs perturbs the electromagnetic field distribution of the sensor. This interaction leads to capacitive and inductive coupling between the sensor and the chip, modeled by the mutual inductance *M* and coupling capacitance $C_{\mathrm{cp}}$.

In Figure 4c, a hardware Trojan is introduced within the chip, modeled by additional parasitic elements $L_{\mathrm{HT}}$, $R_{\mathrm{HT}}$, and $C_{\mathrm{HT}}$. The inclusion of the Trojan alters the overall impedance of the chip, which in turn modifies the electromagnetic coupling with the sensor. This change in the overall circuit manifests as a noticeable variation in the impedance measured at Port 1, enabling the detection of tamper events through shifts in the sensor’s scattering parameters.

### 3.3. Tamper Detection Metric

To distinguish between genuine and tampered signatures, we analyze repeated measurements of $∠S_{11}$ at each frequency point $f_{i}$, using $N_{m}$ measurement samples per frequency. Let ${S_{11}}_{i}^{Gen}$ and ${S_{11}}_{i}^{Tamp}$ denote the random variables corresponding to the reflection coefficient at frequency $f_{i}$ for the genuine and tampered cases, respectively.

Several statistical metrics have been introduced to distinguish the impedance signatures of genuine and Trojanized chips. We employ the most straightforward metric, called the mean difference (MD), as a statistical indicator to distinguish the signatures. MD quantifies the absolute difference between the average reflection responses in the genuine and tampered conditions. The MD is computed for the phase of the reflection coefficient as follows:

(5) $$MD_{\mathrm{Phase}}\left(f_{i}\right)=\left|\mu \left(∠{\left(S_{11}^{\mathrm{Gen}}\right)}_{i}\right)-\mu \left(∠{\left(S_{11}^{\mathrm{Tamp}}\right)}_{i}\right)\right|$$

where μ(·) denotes the mean taken over the $N_{m}$ measurements at each frequency $f_{i}$. Since phase values $∠{S_{11}}_{i}$ are constrained within [−π,π] and exhibit periodic behavior, phase unwrapping is applied to reconstruct a continuous phase response before calculating $MD_{\mathrm{Phase}}\left(f_{i}\right)$.

## 4. Experimental Setup

### 4.1. Simulation Setup

**Figure 5:**
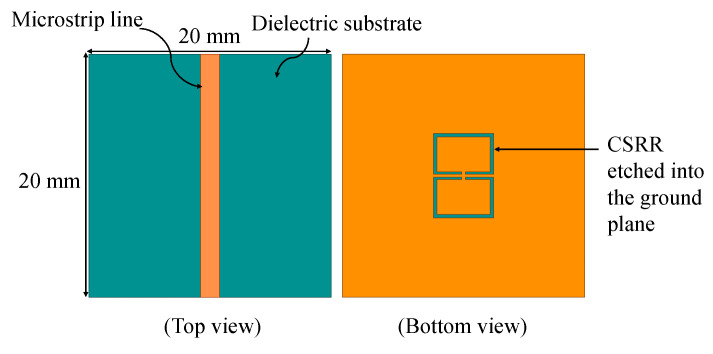
Top and bottom views of a microstrip transmission line loaded with a CSRR sensor.

Figure 5 illustrates the simulated sensor, which consists of a partially etched ground plane forming the sensing area, excited by a microstrip transmission line. The substrate used was a Rogers RO4350B laminate, which has a dielectric constant of 3.48, a loss tangent of 0.0037, and a thickness of 0.76 mm. The microstrip line was designed with a width of 1.4 mm to achieve a characteristic impedance of 50 Ω. Two small rectangular slots were etched into the metallic ground plane to form the complementary resonator structure. To ensure the maximum area of confinement for the electric and magnetic fields, the slots were symmetrically aligned directly beneath the microstrip line. The proposed sensor was simulated using ANSYS HFSS 2025 R1. In our case, the CSRR structure is designed to meet specific requirements: it should produce confined electromagnetic fields to improve sensitivity to local changes, be appropriately sized to cover the chip’s package surface, and demonstrate a sharp resonance with a high quality factor.

### 4.2. Device Under Test (DUT)

For our experiments, we utilized a Digilent Arty S7-50 development board, which is equipped with AMD/Xilinx Spartan-7 XC7S50 FPGAs fabricated using 28 nm CMOS process technology. The FPGA is encapsulated in a 15mm×15mm CSGA324 BGA package. The Arty S7 board supports multiple power delivery networks, such as the core, I/O, and auxiliary voltage networks. The development kit contains a 100 MHz onboard oscillator, capable of generating system clocks via the internal MMCM/PLL resources. The FPGA configuration was carried out using Xilinx Vivado, which was used to synthesize the HDL design, implement the logic on the Spartan-7 device, and generate the bitstream for programming via the board’s JTAG interface.

### 4.3. Measurement Setup

We employed a Mini-Circuits eVNA-63+, a portable vector network analyzer (VNA) capable of operating across a frequency range of 300 kHz to 6 GHz. The VNA includes an internal DC-blocking capacitor, eliminating the need for an external bias tee. We used Mini-Circuits CBL-2FT-SMNM+ shielded precision test cables with male SMA connectors on the DUT side, allowing for a direct connection to the VNA sensor without the need for additional adapters. Calibration was performed precisely up to the SMA interface on the baseboards using the standard open–short–load (OSL) method for one-port reflection ($S_{11}$) and impedance measurements. The measurements were conducted over a frequency range of 3.5 GHz to 5 GHz, using 1000 evenly spaced sampling points to ensure high spectral resolution. This band was selected because the resonance frequency of the CSRR sensor lies within this range, where variations due to external influences are most pronounced and detectable. The VNA was configured with a 10 kHz intermediate frequency (IF) bandwidth and an output power level of 10 dBm.

**Figure 6:**
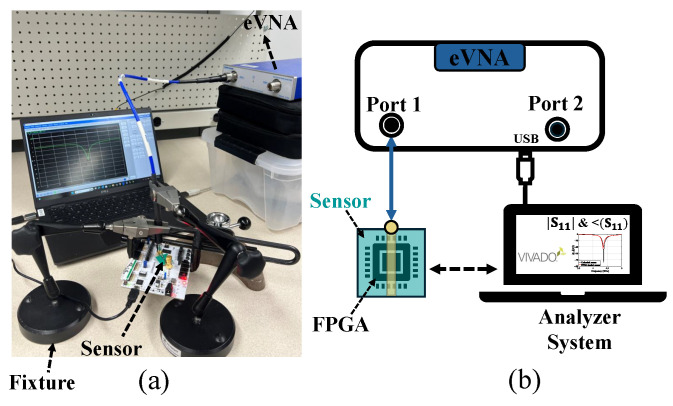
(**a**) The measurement setup consists of an eVNA capturing $S_{11}$ traces, fixtures to maintain a constant vertical distance between the sensor and the chip, and a CSRR sensor positioned directly above the chip. (**b**) Experimental setup diagram.

The sensor is connected to the eVNA through the test cable. We automated the measurement process to program the FPGA and performed 500 repeated $S_{11}$ measurements for each experiment, which were then stored on the analyzer system. To ensure the experiment is repeatable, the sensor must be positioned and aligned precisely in the same location above the FPGA for each measurement. To maintain consistent and accurate spacing between the sensor and the chip under test, two mechanical fixtures were used to securely hold the CSRR sensor at a fixed distance above the chip, as illustrated in Figure 6. This controlled placement is critical for maintaining stable measurements and reproducible sensor responses.

For optimal sensitivity, the DUT must be located within the near-field region of the sensor, where electromagnetic fields are predominantly reactive and strongly concentrated near the sensor, enabling strong capacitive and inductive coupling. Given that the CSRR sensor used in this work resonates at 4.37 GHz, the corresponding free-space wavelength is

(6) $$\lambda =\frac{c}{f}=\frac{3\times 10^{8}\;m/s}{4.37\times 10^{9}\;\mathrm{Hz}}\approx 68.6\;\mathrm{mm}.$$

The boundary of the near-field region for a non-radiating structure, such as a CSRR, is typically approximated by [38]

(7) $$r_{\mathrm{near}}≲\frac{\lambda }{2\pi }\approx \frac{68.6\;\mathrm{mm}}{2\pi }\approx 10.9\;\mathrm{mm}.$$

On the other hand, the chip and its package materials exhibit a nonzero loss tangent, which introduces dielectric losses when placed too close to the sensor. These losses dissipate electromagnetic energy, resulting in a reduction in the sensor’s quality factor and broadening the $S_{11}$ resonance, which in turn reduces its ability to detect subtle changes. Thus, the sensor-to-chip spacing must be carefully optimized. In other words, it must close enough to ensure effective near-field interaction, yet far enough to avoid excess energy loss and preserve the resonance sharpness.

Through empirical evaluation, a separation distance of 3 mm was found to provide an effective balance between strong electromagnetic coupling and minimal degradation of the resonance characteristics, ensuring both sensitivity and measurement stability. According to the eVNA Mini-Circuits datasheet [39], a practical phase resolution of 0.001 to 0.01 degrees can be achieved when using a 1 Hz IF bandwidth, demonstrating the high phase resolution and stability of the system. This confirms the setup’s capability to reliably capture even subtle changes in the sensor’s phase response.

## 5. Results

### 5.1. Case Studies of Tampering Detection Using CSRR Sensor

There are an unlimited number of possibilities for tamper events, and naturally it is not feasible to cover them all. However, we select a representative set of tamper events that span different tamper categories to demonstrate the capability of our method in addressing a range of threats. Our approach is transferable to other classes of tampering, as long as they result in measurable changes to the PDN’s impedance. To evaluate the sensor’s ability to detect changes in the die’s PDN impedance, we followed a step-by-step approach, beginning with more pronounced modifications. We began by comparing the powered-off and powered-on states, then transitioned to the unconfigured versus configured states of the chip, and subsequently tested circuits of varying sizes. This gradual progression allowed us to assess whether the sensor could reliably detect increasingly smaller variations. Ultimately, we applied the method to detect subtle changes, such as those introduced by hardware Trojans and changes in placement and routing.

#### 5.1.1. Baseline Experiment: Effect of Passive DUT on the Sensor’s Behavior

**Figure 7:**
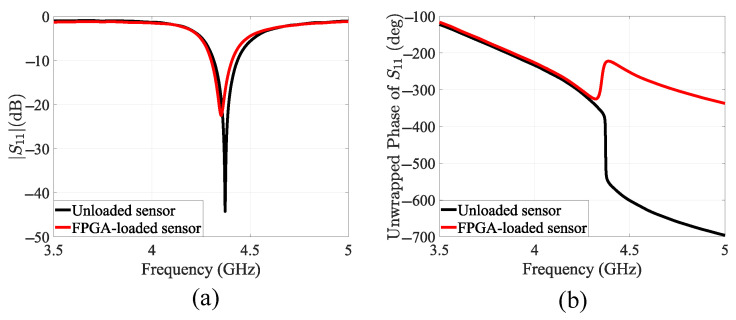
(**a**) Measured $|S_{11}|$ for the unloaded (bare) sensor and the FPGA-loaded sensor. (**b**) Measured phase of $S_{11}$ for the unloaded sensor and the FPGA-loaded sensor.

In the first experiment, the FPGA was powered off and placed in close proximity to the sensor. As shown in Figure 7, a significant change was observed in both the magnitude and phase of the sensor’s $S_{11}$ parameter. In the absence of the chip, the sensor exhibits a distinctive $S_{11}$ response with a high quality factor and sharp resonance at 4.37 GHz. When the chip is placed within the sensor’s near-field region, the response changes noticeably, with the resonance shifting to 4.35 GHz and a reduced quality factor. This shift is caused by the introduction of a new material, the combined package and die of the FPGA, which has different permittivity and permeability compared to air. Since the difference in impedance between air and the DUT is substantial, the resulting shift in the sensor’s response is clearly visible.

#### 5.1.2. Case Study 1: Influence of FPGA State on Sensor Impedance Characteristics

**Figure 8:**
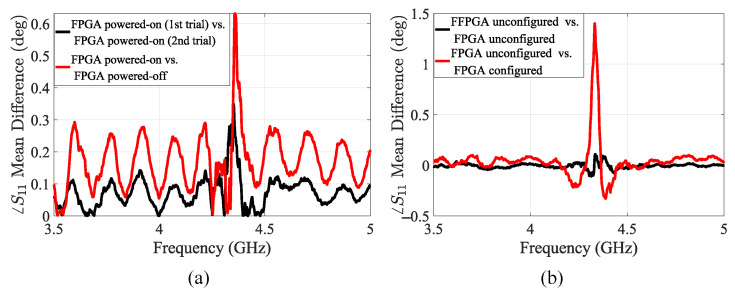
(**a**) The mean phase difference in the sensor’s reflection response in the case of powering on and off the FPGA. (**b**) The mean phase difference in the sensor’s reflection response for the configuration mode of the FPGA.

This case study examines how the global state of the FPGA affects the impedance response observed at the sensor. We analyzed the scattering parameters of the sensor under two primary FPGA conditions: powered off and powered on without configuration. In the powered-on state, the FPGA is supplied with a nominal voltage but has not yet been configured with a bitstream. In this case, a noticeable shift in the scattering parameter signature emerged around the resonance frequency of the sensor (4.37 GHz). This indicates that powering on the FPGA activates internal transistors, effectively adding capacitance from gate channels and resistance from conducting paths to the circuit. These additional components reshape the RLC network, changing the overall impedance and modifying its frequency response, which in turn affects the sensor’s impedance profile, see Figure 8a.

We further compared the unconfigured powered-on state of the FPGA to the case where it was configured with an AES-128 encryption IP core. In the unconfigured state, the chip is ready to be configured, and the impedance of the circuit responsible for configuring the FPGA is active. After the FPGA has been configured, various components of the circuit, including logic blocks, interconnects, and I/O buffers, are activated, and a distinct resistive and capacitive impedance signature is observed. These changes alter the electromagnetic field distribution around the sensor and lead to observable shifts in the sensor’s $∠S_{11}$ response, as shown in Figure 8b.

#### 5.1.3. Case Study 2: Influence of Circuit Size on Sensor Impedance Characteristics

In this case study, we investigated how the size of a digital circuit implemented on the FPGA affects the impedance profile observed by an external sensor. The objective is to determine whether varying the circuit size leads to measurable changes in the sensor’s reflection response, enabling passive detection of internal FPGA activity. To analyze this, we synthesized chains of NOT gates in Vivado, varying the number of gates (Lookup Tables on FPGA) across three configurations: 100, 10,000, and 20,000 gates. As the number of gates increases, the overall circuit size grows proportionally. This increase introduces more active logic elements and interconnects into the FPGA circuitry, thereby altering both the static electric field emanated from the chip and the impedance of the chip’s PDN. These changes modify the near-field distribution around the sensor and affect the measured $∠S_{11}$ response.

**Figure 9:**
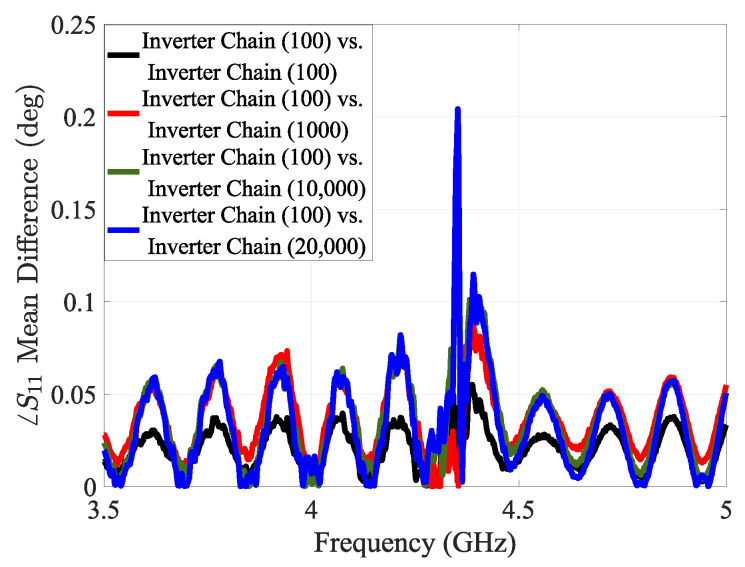
The mean phase difference in the sensor’s reflection response for different sizes of the NOT gate chain.

The experimental results highlight that circuit size (independent of functionality) has a measurable impact on the PDN impedance of the FPGA, as seen from the sensor’s perspective, see Figure 9.

This sensitivity may be exploited to infer information about the internal resource utilization of the chip, even without activating/triggering the circuit.

#### 5.1.4. Case Study 3: Hardware Trojan Detection

To evaluate the effectiveness of the proposed detection method in identifying small, inactive hardware modifications, we conducted a set of experiments focused on detecting dormant hardware Trojans (HTs), which are a particularly challenging class of malicious alterations due to their stealthy behavior.

For this purpose, we implemented Trojan-free and Trojan-inserted circuits and tried to detect them by analyzing the reflection response from the sensor above the configured chip. Importantly, in all experiments, the HTs remained inactive, and the circuit operated in an idle state. This setup was designed to validate the method’s applicability to dormant HTs.

**Figure 10:**
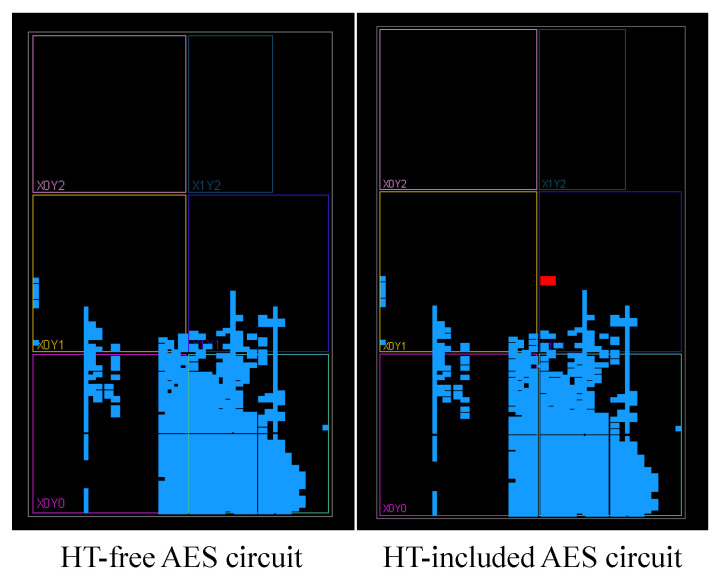
FPGA implementation of AES circuits: (**left**) HT-free design and (**right**) HT-inserted version. Blue cells represent the standard AES logic blocks, while red cells indicate inserted hardware Trojan.

We used the AES-T100 benchmarks from Trust-Hub [40], which provide register-transfer level (RTL) HT implementations embedded in an AES-128 encryption core. The original design is an 11-stage pipelined AES IP that processes 128-bit data blocks through ten rounds of encryption. A major challenge in HT detection is achieving stealthy integration during implementation. When designs containing Trojans are compiled with default placement and routing settings, synthesis tools like Vivado often cause extensive layout changes, making the comparison challenging. To address this, we used a method that accounts for stealthy behavior. After compiling the HT-inserted design and fixing the placement of all cells and logic elements, we manually removed the Trojan logic and its connections to create a Trojan-free version. This ensures the original placement and routing are maintained, enabling a fair and realistic comparison. Figure 10 presents an example of the resulting designs. It highlights in blue the genuine (HT-free) AES logic elements, offering a visual comparison that underscores the structural differences between the two implementations.

**Figure 11:**
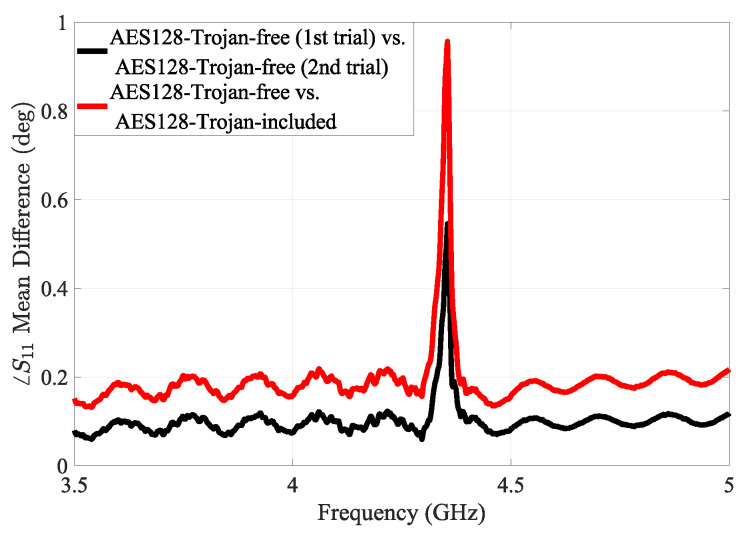
The mean phase difference in the sensor’s reflection response in the case of HT-free and HT-included AES implementation.

Figure 11 shows the mean phase differences in the sensor’s measured reflection responses for the AES-T100 Trojan case, highlighting the method’s effectiveness in detecting subtle variations in impedance and electric field modification induced by the trigger configuration of dormant hardware Trojans. While we used this specific hardware Trojan, the proposed detection method is broadly applicable to other HT benchmarks.

#### 5.1.5. Case Study 4: Effect of Changing the Placement and Routing

**Figure 12:**
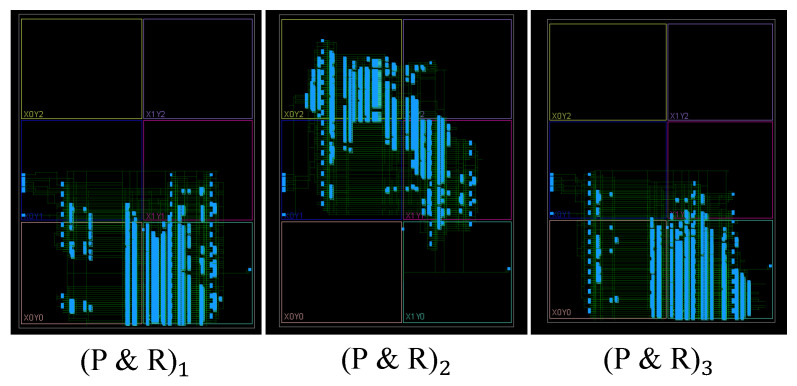
Change in P&R in a genuine AES layout on the FPGA, blue cells represent the standard AES logic blocks.

For the next case study, we considered a tamper event that alters the placement and routing (P&R) of the design. To isolate the effect of such modifications, we kept the circuit size and logic elements unchanged, focusing solely on changes in placement and routing to evaluate the efficacy of our method in detecting this class of tampering. We show these routings in the FPGA layout using the Vivado design tool, as illustrated in Figure 12.

In an FPGA, placement and routing determine the physical layout and interconnection of logic elements. Even when the logic function remains the same, altering P&R changes the length, topology, and load distribution of interconnects across the chip. These routing resources introduce parasitic capacitances, resistances, and inductances, which affect the overall PDN impedance.

**Figure 13:**
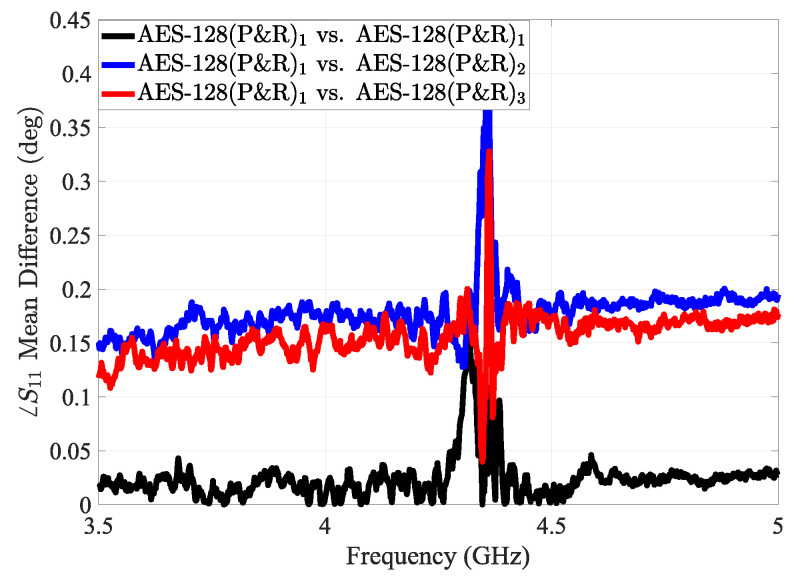
The mean phase difference in the sensor’s reflection response in the case of different routing of the AES implementation.

As a result, different P&R configurations lead to measurable changes in the PDN impedance profile of the FPGA, especially in the high-frequency domain, where parasitic effects are dominant. These variations in impedance, in turn, perturb the near-field electromagnetic distribution of the sensor and change its scattering parameter. Figure 13 shows the mean phase difference in the sensor’s reflection response in the case of different routing of the AES implementation.

## 6. Discussion and Future Work

### 6.1. Performance Comparison with a Commercial Probe

Commercial magnetic near-field probes function based on Faraday’s law, detecting the magnetic (H) field near a PCB or chip by using a loop antenna typically realized as a small shielded loop structure. These probes convert magnetic flux variations into a voltage that is measured and are often shielded to suppress the influence of electric fields, enhancing selectivity and reducing measurement artifacts. The loop’s size determines a trade-off between spatial resolution and sensitivity: smaller loops provide more precise localization but lower signal strength. To boost sensitivity, some commercial probes integrate a low-noise amplifier (LNA), forming active probes. These probes are used in pre-compliance EMC testing, electromagnetic interference (EMI) diagnosis, and source localization, enabling engineers to non-invasively scan a board for high-current traces and radiating structures. To detect tampering using these probes, measurements must typically be taken point by point to gather information across the entire area. In contrast, our sensor can monitor a single trace in a controlled environment and infer tampering across the entire chip. Additionally, while some of these probes require physical contact with the surface, our sensor operates in a non-contact manner. Another key advantage is cost; commercial probes are often expensive, whereas our sensor offers a low-cost alternative. In [2], a novel near-field backscattering sensing method for hardware Trojan detection in integrated circuits is introduced. By combining custom-designed E- and H-field probes, they excite a carrier signal into the device and capture the modulated back-scattered emissions, which carry unique signatures of the underlying logic circuit. Their approach achieves high spatial resolution (1 mm) and successfully detects dormant hardware Trojans with 100% accuracy and no false positives in controlled FPGA experiments. This approach presents several challenges. It depends on the ability to inject a clean carrier signal into the device, which may not always be feasible in commercial or secured hardware environments. The backscattered signals are inherently weak and susceptible to interference from environmental noise and other circuit activities, requiring careful calibration and high-sensitivity RF equipment. Furthermore, although the technique demonstrates acceptable results in controlled experiments, scaling it for widespread industrial adoption faces significant hurdles due to the complexity of probe alignment, the need for localized scanning, and the difficulty of automating the measurement process. Table 1 provides a comparison between the proposed tamper detection method and existing EM-based sensing techniques in the literature in terms of system compatibility, implementation complexity, cost, and the requirement for Trojan activation. In contrast to prior approaches that require Trojan activation or internal access to the chip, this work demonstrates a passive, low-cost detection method based on external impedance sensing. The proposed technique is fully compatible with legacy systems and does not require hardware modification, offering a practical solution for scalable, non-invasive tamper detection.

### 6.2. Accuracy and Sensitivity Considerations of the CSRR Sensor

In material characterization, where parameters such as permittivity or permeability are known and controllable, the sensitivity of a CSRR sensor is often defined as the shift in its resonance frequency or change in quality factor in response to variations in the permittivity or permeability of the material under test. However, in our case, the sensor is intended for tamper detection, where an attack introduces unknown and unpredictable physical changes to the die, resulting in changes to its equivalent impedance. Since the resulting variations in impedance cannot be directly measured, it is not straightforward to quantify the sensitivity of the measurement setup in absolute terms. Therefore, we define sensitivity in terms of the corresponding changes observed in the $S_{11}$ parameter. Improving the sensitivity of a CSRR-based sensor requires enhancing the degree to which its resonance frequency and quality factor respond to small variations in the properties of the MUT. In the current work, the sensor was designed as a proof of concept, but its sensitivity can be significantly improved through careful design choices.

A key factor in increasing sensitivity is strengthening the localized electromagnetic near fields around the CSRR structure. This can be achieved by modifying the resonator geometry to increase its effective capacitance and inductance, thereby enhancing its ability to store electromagnetic energy. For instance, implementing a meandered, multi-ring, or fractal CSRR can increase the reactive energy storage and field confinement, making the sensor more responsive to small impedance changes.

Another effective strategy involves selecting the appropriate substrate materials for the sensor. Using a high-permittivity substrate enhances electric field confinement around the sensor, increasing its interaction with the nearby environment. Simultaneously, employing low-loss substrates improves the quality factor of the resonance, resulting in sharper resonance dips in the scattering parameter response, which improves the detectability of small impedance changes.

Sensor size and operational frequency also play critical roles. In our design, the CSRR was slightly larger than the chip area, enabling single-trace detection in a controlled environment. However, designing a smaller sensor would result in a higher resonance frequency, which provides greater spatial resolution due to stronger and more localized fields. However, this comes at the cost of shallower field penetration into the chip, greater radiation losses, and generally a lower *Q* factor. Additionally, smaller sensors may require scanning across the chip surface, making detection a multi-step process. Conversely, a larger sensor operating at lower frequencies produces weaker but deeper penetrating fields, making it more suitable for detecting attacks embedded deeper within the chip or package layers. Hence, there is a fundamental trade-off between resolution and penetration depth, and the optimal sensor design must align with the spatial scale and depth of the tamper event to be detected.

Most importantly, the resonance frequency of the sensor should be aligned with the frequency range in which the impedance of the target system exhibits significant variation. In our case, since the goal was to detect chip-level tampering, which occurs at GHz frequencies due to changes in on-chip interconnects and active circuit states, we designed the sensor to operate within this range and to cover the entire chip area.

For package-level or PCB-level attacks, where the impedance changes are distributed over larger structures and occur at lower frequencies, a lower-resolution sensor operating at lower frequencies would be more appropriate and effective.

Finally, one effective solution to reduce noise and improve sensitivity could be the integration of an LNA with the CSRR sensor. The LNA boosts weak reflected signals and enhances the signal-to-noise ratio. However, proper frequency matching between the CSRR and LNA, along with careful low-noise circuit design, is essential.

### 6.3. Future Works

Future improvements to the proposed sensing framework may focus on enhancing accuracy. The sensing resolution can be increased by designing more advanced CSRR geometries that improve electromagnetic field confinement and sensitivity to impedance variations. Another interesting future direction is the application of the proposed sensor to side-channel attacks on cryptographic hardware, similar to what has been presented in [32]. By exploiting data-dependent impedance variations in the chip’s power delivery network, the sensor could potentially be used to extract secret keys without requiring physical contact or activation of the cryptographic logic.

## 7. Conclusions

In this work, we introduced a contactless method for detecting chip-level tampering by repurposing the operating principle of a complementary split-ring resonator (CSRR), originally developed for material characterization. Any tampering event can alter the impedance of the chip’s PDN. By placing the chip within the sensitive electromagnetic near-field region of the sensor, these impedance changes alter the local field environment, resulting in measurable variations in the sensor’s reflection parameter response. This enables reliable detection of a broad range of tamper scenarios without the need to activate any part of the circuit or modify the FPGA design. Through extensive experiments across multiple FPGA implementations, we showed that even subtle changes, such as those caused by small, dormant hardware Trojans or modifications to placement and routing, produce measurable effects on the sensor’s response. Using a simple statistical measure, the mean difference (MD), we confirmed that these events can be detected with high confidence, validating the effectiveness and practicality of the proposed approach.

## Figures and Tables

**Table 1:** Comparison of EM-based trojan detection methods.

Detection Technique (Ref.)	Legacy Compatible	Chip Access/Connection	Complexity and Cost	Trojan Activation
[External probe [41]]	Yes	No	Moderate	Yes
[External probe [42]]	Yes	No	Moderate	No
[External probe [43]]	Yes	No	Moderate	No
[External probe [44]]	Yes	No	Moderate	Yes
[External Probe [2]]	Yes	No	High	No
[On-Chip sensor [45]]	No	Yes	High	Yes
[[On-Chip sensor [46]]	No	Yes	High	Yes
[[On-Chip sensor [47]]	No	Yes	High	Yes
[External sensor [31]]	Yes	Yes	Low	No
[This Work]	Yes	No	Low	No

## Data Availability

The original contributions presented in this study are included in the article. Further inquiries can be directed to the corresponding author(s).
